# Prevalence of Anti-Tuberculosis Drug Resistance in Foreign-Born Tuberculosis Cases in the U.S. and in Their Countries of Origin

**DOI:** 10.1371/journal.pone.0049355

**Published:** 2012-11-07

**Authors:** Allison B. Taylor, Ekaterina V. Kurbatova, J. Peter Cegielski

**Affiliations:** Division of Tuberculosis Elimination, National Center for HIV/AIDS, Viral Hepatitis, STD, and TB Prevention, the U.S. Centers for Disease Control and Prevention, Atlanta, Georgia, United States of America; McGill University, Canada

## Abstract

**Background:**

Foreign-born individuals comprise >50% of tuberculosis (TB) cases in the U.S. Since anti-TB drug resistance is more common in most other countries, when evaluating a foreign-born individual for TB, one must consider the risk of drug resistance. Naturally, clinicians query The Global Project on Anti-tuberculosis Drug Resistance Surveillance (Global DRS) which provides population-based data on the prevalence of anti-TB drug resistance in 127 countries starting in 1994. However, foreign-born persons in the U.S. are a biased sample of the population of their countries of origin, and Global DRS data may not accurately predict their risk of drug resistance. Since implementing drug resistance surveillance in 1993, the U.S. National TB Surveillance System (NTSS) has accumulated systematic data on over 130,000 foreign-born TB cases from more than 200 countries and territories. Our objective was to determine whether the prevalence of drug resistance among foreign-born TB cases correlates better with data from the Global DRS or with data on foreign-born TB cases in the NTSS.

**Methods and Findings:**

We compared the prevalence of resistance to isoniazid and rifampin among foreign-born TB cases in the U.S., 2007–2009, with US NTSS data from 1993 to 2006 and with Global DRS data from 1994–2007 visually with scatterplots and statistically with correlation and linear regression analyses. Among foreign-born TB cases in the U.S., 2007–2009, the prevalence of isoniazid resistance and multidrug resistance (MDR, i.e. resistance to isoniazid and rifampin), correlated much better with 1993–2006 US surveillance data (isoniazid: *r* = 0.95, *P*<.001, MDR: *r* = 0.75, *P*<.001) than with Global DRS data, 1994–2007 (isoniazid: *r* = 0.55, *P* = .001; MDR: *r* = 0.50, *P*<.001).

**Conclusion:**

Since 1993, the US NTSS has accumulated sufficient data on foreign-born TB cases to estimate the risk of drug resistance among such individuals better than data from the Global DRS.

## Introduction

Since 2001, over half of the tuberculosis (TB) cases in the United States (U.S.) have occurred in people who were born outside the U.S. and the prevalence of drug resistance is up to 6-fold higher among foreign-born than U.S.-born TB cases [1], [2]. Because conventional drug susceptibility tests (DST) typically take 3 weeks to 3 months, TB disease is generally treated empirically until DST results become available. Latent TB infection (LTBI) is always treated empirically, generally with isoniazid alone. Since anti-TB drug resistance is more common in most other countries, when evaluating a foreign-born individual for TB, one must consider the risk of drug resistance.

The World Health Organization (WHO)/International Union Against Tuberculosis and Lung Diseases (IUATLD) Global Project on Anti-Tuberculosis Drug Resistance Surveillance (DRS) (Global DRS) reported that among all incident TB cases in 2008 globally, 3.6% are estimated to have multidrug-resistant (MDR) TB, defined as TB caused by strains of *Mycobacterium tuberculosis* (MTB) that are resistant to at least isoniazid (INH) and rifampin (RIF) [3]. Global DRS data may be used to guide evaluation and management of foreign-born TB cases in the U.S. because they provide population-based estimates of the patterns of drug resistance in the countries where the Global DRS has been carried out. The Global DRS, however, may not be the best source of information because foreign-born individuals diagnosed with TB in the U.S. are not a representative sample of TB patients in the country of origin, so the prevalence of drug resistance may differ.

The U.S. National TB Surveillance System (NTSS) has collected national TB incidence data from 1953, and has included DST results since 1993, when universal drug susceptibility testing and drug resistance surveillance was implemented [1]. We hypothesized that prevalence of drug resistance among foreign-born TB cases in the U.S. would be better predicted by the prevalence of drug resistance in previous foreign-born cases in the U.S. from the same country or region from NTSS data than by the prevalence of drug resistance among TB cases in their countries of origin based on Global DRS data.

## Methods

### Population

The methodology and results of the Global DRS have been published in 4 volumes reporting in total surveys (or surveillance data) from 114 different countries carried out 1994–96 (35 countries or subnational geographic regions of large countries), 1997–99 (58 countries/regions), 2000–02 (77 countries/regions), and 2002–07 (81 countries/regions) [4]–[7]. The fifth volume of the Global DRS results covering 2008–2010 did not have sufficient detail to be included in this analysis [3]. Many countries were surveyed more than once. Global DRS data provide population-based representative estimates of the prevalence of drug resistance among pulmonary TB cases separately for new and for previously treated cases.

The U.S. national surveillance system includes data on all verified TB cases reported to the Centers for Disease Control and Prevention (CDC) and counted starting in 1993 [1]. We selected all pulmonary TB cases among individuals who were born outside the U.S. but diagnosed with TB while living in the U.S. and reported to the CDC. In most countries, case detection is based on microscopy, and Global DRS data include only sputum smear-positive cases [7]. For these countries, we limited the corresponding U.S. data to sputum smear-positive cases. In Europe and a few other countries where mycobacterial cultures are routine, and case detection is based on sputum culture, the Global DRS data were not limited to smear-positive cases [7]. For these countries, we did not limit the U.S. data based on sputum smear results either. Since sample size calculations in Global DRS are based on expected prevalence among new TB cases, we limited our analysis of both data sets to new cases with no previous history of TB.

### Definitions

We focused our analysis on prevalence of isoniazid resistance because LTBI is usually treated with isoniazid alone, and on multidrug resistance because MDR TB requires different treatment than drug-susceptible TB. For both surveillance systems, the prevalence of isoniazid resistance was calculated as the number of isoniazid-resistant isolates divided by the number of isolates tested against at least isoniazid. The prevalence of MDR TB was calculated as the number of isolates resistant to at least isoniazid and rifampin divided by the total number of isolates tested against at least isoniazid and rifampin.

### Data Analysis

We grouped all TB cases in NTSS by country of origin and determined how many culture-positive cases a particular country contributed to the foreign-born case count. For NTSS data, we aggregated cases into two time periods: “recent” cases, 2007–2009, and “previous” cases, 1993–20062.

We visually assessed scatterplots to assess linear relationships between prevalence of drug resistance in recent NTSS and previous NTSS data and between recent NTSS and Global DRS data. We conducted correlation analysis using the Spearman coefficient for non-parametric correlations to assess the direction, strength and significance of linear relationships. We correlated by country or region of origin the prevalence of drug resistance among U.S. foreign-born cases in recent NTSS data (2007–2009), with the prevalence of drug resistance among U.S. foreign-born cases in the corresponding NTSS data for the earlier period (1993–2006), and with the prevalence of drug resistance in those same countries or regions in the Global DRS data for combined previous periods (1994–2007). We used simple linear regression to determine if the prevalence of drug resistance by country or region in recent NTSS data is better predicted by previous NTSS data or by Global DRS data. We estimated a slope of the regression line and reported R^2^ for magnitude of effect and *P* value of F test for overall significance of linear regression model.

To determine the consistency of these correlations over time, we subdivided the previous cases in the NTSS data into 4 time periods (1993–1996, 1997–1999, 2000–2002, and 2003–2006) corresponding most closely to the 4 waves of the Global DRS. Then we repeated the analysis separately for each time period, in each case correlating the prevalence of drug resistance by country or region in each period of the US data and in each wave of the Global DRS data as the predictor variable with the prevalence in the next period of U.S. data as the dependent variable. To ensure stable proportions for the U.S. foreign-born cases, if a country contributed more than 100 culture-positive TB cases in all five defined time periods, the country itself was the unit of analysis, otherwise the country was grouped into one of nine WHO-defined TB epidemiologic regions (**Table 1**) [8]. Although NTSS reported >100 TB cases in the U.S. for Haiti, Laos, and Pakistan for all time periods, we added these cases to the respective WHO regions as no Global DRS data were available for these countries. We excluded from analysis NTSS TB cases listed as foreign born but with place of birth in the territories of the U.S. and cases with no specified country of origin.

**Table 1:** Isoniazid Resistant and Multidrug-Resistant Tuberculosis Among Foreign-born*,† Cases in Recent U.S. NTSS (2007–2009), Previous U.S. NTSS (1993–2006) and Global DRS (1994–2007) Data.

Country or region	Country’s rank in NTSS ‡	All with DST			INH resistant						MDR					
		NTSS (2007–2009)	NTSS (1993–2006)	Global DRS (1994–2007)	NTSS (2007–2009)		NTSS (1993–2006)		Global DRS		NTSS (2007–2009)		NTSS (1993–2006)		Global DRS (1994–2007)	
		N	N	N	n	%	n	%	n	%	n	%	n	%	n	%
**Countries**																
**Peru**	13	154	873	5,188	28	18.2	150	17.2	495	9.5	9	5.8	43	4.9	189	3.6
**Dominican Republic**	16	96	647	303	14	14.6	115	17.8	60	19.8	4	4.2	40	6.2	20	6.6
**Viet Nam**	3	609	2,900	2,259	117	19.2	590	20.3	438	19.4	14	2.3	38	1.3	59	2.6
**Ethiopia**	8	152	521	804	15	9.9	53	10.2	62	7.7	3	2.0	5	1.0	13	1.6
**Rep of Korea**	10	206	1,212	7,492	28	13.6	171	14.1	657	8.8	4	1.9	36	3.0	162	2.2
**India**	4	433	1,764	3,562	51	11.8	217	12.3	441	12.4	8	1.8	50	2.8	82	2.3
**China**	5	390	1,611	10,004	30	7.7	180	11.2	1,489	14.9	6	1.5	40	2.5	572	5.7
**Guatemala**	6	324	947	668	20	6.2	80	8.4	72	10.8	5	1.5	10	1.1	20	3.0
**Philippines**	2	839	3,495	965	131	15.6	568	16.3	130	13.5	12	1.4	74	2.1	39	4.0
**Mexico**	1	2,404	10,758	334	189	7.9	999	9.3	24	7.2	24	1.0	183	1.7	8	2.4
**Cambodia**	15	102	470	638	8	7.8	48	10.2	41	6.4	1	1.0	5	1.1	0	0
**Ecuador**	14	132	767	812	5	3.8	63	8.2	89	11.0	1	0.8	12	1.6	40	4.9
**Honduras**	9	246	716	626	7	2.8	33	4.6	47	7.5	1	0.4	3	0.4	11	1.8
**El Salvador**	12	162	733	611	7	4.3	59	8.0	8	1.3	0	0	8	1.1	2	0.3
**WHO-defined TB epidemiological regions** §																
**Eastern Europe**	-	150	787	16,660	27	18.0	106	13.5	4,040	24.2	14	9.3	29	3.7	1717	10.0
**South-East Asia**	-	279	915	7,599	43	15.4	136	14.9	612	8.1	13	4.7	36	3.9	131	1.7
**Western Pacific**	-	240	1,267	13,363	26	10.8	158	12.5	794	5.9	8	3.3	42	3.3	132	1.0
**Eastern Mediterranean**	-	239	996	4,406	23	9.6	110	11.0	310	7.0	5	2.1	16	1.6	90	2.0
**Africa – low HIV**	-	141	616	3,227	16	11.3	70	11.4	210	6.5	2	1.4	11	1.8	26	0.8
**Latin America**	-	504	3,268	12,609	56	11.1	361	11.0	639	5.1	5	1.0	53	1.6	135	1.1
**Africa – high HIV**	-	243	952	12,852	13	5.3	80	8.4	875	6.8	1	0.4	12	1.3	224	1.7
**Established Market Economies**	-	133	1,008	51,458	6	4.5	47	4.7	2,854	5.5	0	0	6	0.6	521	1.0
**Central Europe**	-	133	771	15,575	3	2.3	33	4.3	397	2.5	0	0	5	0.6	88	0.6
**TOTAL**	-	8,311	37,994	172,015	863	10.4	4,427	11.7	14,784	8.6	126	1.5	728	1.9	4281	2.5

**Note.** Countries and regions are sorted in descending order by prevalence of MDR TB in recent U.S. NTSS data (2007–2009).

N = total number of patients from specific country or region with reported DST results.

n = total number of patients from specific country or region with reported pattern of resistance (INH resistant or MDR, respectively).

NTSS = National Tuberculosis Surveillance System.

Global DRS =  Global Project on Anti-Tuberculosis Drug Resistance Surveillance.

* Foreign-born include persons born outside the United States, American Samoa, the Federated States of Micronesia, Guam, the Republic of the Marshall Islands, Midway Island, the Commonwealth of the Northern Mariana Islands, Puerto Rico, the Republic of Palau, the U.S. Virgin Islands, and U.S. minor and outlying Pacific islands.

† TB cases from 14 countries for which prevalence of drug-resistant TB was reported separately were not included in respective WHO regions counts.

‡ Rank of country was assigned based on 5-year average number of TB cases from that country in the U.S. in 2005–2009. [1].

§ WHO nine epidemiological regions [8] include following countries:

**Africa – high HIV:** Botswana, Burkina Faso, Burundi, Cameroon, Central African Rep, Chad, Congo, Côte d’Ivoire, DR Congo, Ethiopia, Equatorial Guinea, Gabon, Kenya, Lesotho, Malawi, Mozambique, Namibia, Nigeria, Rwanda, South Africa, Swaziland, Uganda, UR Tanzania, Zambia, Zimbabwe. Ethiopia was excluded from the region estimates and reported separately.

**Africa – low HIV:** Algeria, Angola, Benin, Cape Verde, Comoros, Eritrea, Gambia, Ghana, Guinea, Guinea-Bissau, Liberia, Madagascar, Mali, Mauritania, Mauritius, Niger, Sao Tome & Principe, Senegal, Seychelles, Sierra Leone, Togo.

**Central Europe:** Albania, Bosnia & Herzegovina, Croatia, Cyprus, Hungary, Poland, Serbia & Montenegro, Slovakia, Slovenia, TFYR Macedonia, Turkey.

**Eastern Europe:** Armenia, Azerbaijan, Belarus, Bulgaria, Estonia, Georgia, Kazakhstan, Kyrgystan, Latvia, Lithuania, Rep Moldova, Romania, Russian Federation, Tajikistan, Turkmenistan, Ukraine, Uzbekistan.

**Eastern Mediterranean:** Afghanistan, Bahrain, Djibouti, Egypt, Iran, Iraq, Jordan, Kuwait, Lebanon, Libyan Arab Jamahiriya, Morocco, Oman, Pakistan, Qatar, Saudi Arabia, Somalia, Sudan, Syrian Arab Rep, Tunisia, United Arab Emirates, West Bank & Gaza Strip, Yemen.

**Established Market Economies**: Andorra, Australia, Austria, Belgium, Canada, Czech Rep, Denmark, Finland, France, Germany, Greece, Iceland, Ireland, Israel, Italy, Japan, Luxembourg, Malta, Monaco, Netherlands, New Zealand, Norway, Portugal, San Marino, Singapore, Spain, Sweden, Switzerland, Vatican City, United Kingdom.

**Latin America:** Anguilla, Antigua & Barbuda, Argentina, Bahamas, Barbados, Belize, Bermuda, Bolivia, Brazil, British Virgin Is, Cayman Is, Chile, Colombia, Costa Rica, Cuba, Dominica, Dominican Republic, Ecuador, El Salvador, Grenada, Guatemala, Guyana, Haiti, Honduras, Jamaica, Mexico, Montserrat, Netherlands Antilles, Nicargua, Panama, Paraguay, Peru, St Kitts & Nevis, St Lucia, St Vincent & the Grenadines, Suriname, Trinidad & Tobago, Turks & Caicos Is, Uruguay, Venezuela. Dominican Republic, Ecuador, Guatemala, El Salvador, Honduras, Mexico and Peru were excluded from the region estimates and reported separately.

**South-East Asia:** Bangladesh, Bhutan, DPR Korea, India, Indonesia, Maldives, Myanmar, Nepal, Sri Lanka, Thailand, Timor-Leste. India was excluded from the region estimates and reported separately.

**Western Pacific:** Brunei Darussalam, Cambodia, China, China Hong Kong SAR, China Macao SAR, Cook Is, Fiji, French Polynesia, Kiribati, Lao PDR, Malaysia, Mongolia, Nauru, New Caledonia, Niue, Papua New Guinea, Philippines, Rep Korea, Solomon Is, Taiwan, Tokelau, Tonga, Tuvalu, Vanuatu, Viet Nam, Wallis & Futuna Is, Western Samoa. Cambodia, China, Philippines, Republic of Korea and Viet Nam were excluded from the region estimates and reported separately.

A categorical trend analysis of prevalence of drug resistant TB in the U.S. foreign-born TB cases was conducted assessing univariate trend across five time periods using Cochran-Armitage trend test. Statistical analyses were performed using SAS software, version 9.1 (SAS Institute Inc., Cary, NC). A *P* value<.05 was considered statistically significant.

## Results

From 1 January 1993 through 31 December 2009, a total of 130,672 foreign-born TB cases from more than 200 countries were reported in the U.S. NTSS. Of these, 103,978 (79.6%) were culture positive, and 100,768 (96.9%) had DST results reported. Of 29,904 without DST results, 26,694 (89.3%) were from culture-negative cases, and 26,848 (89.8%) of 29,904 were treated initially with isoniazid and rifampin.

Of 130,672 foreign-born TB cases, 94,683 (72.5%) had previously untreated pulmonary TB. Any resistance to isoniazid was reported in 8,424 (11.2%) of 75,208 of these new, pulmonary TB cases with available DST results for at least isoniazid. MDR TB was reported in 1,292 (1.7%) of 75,113 cases whose cultures were tested against at least isoniazid and rifampin. The overall prevalence of drug-resistance among foreign-born TB cases decreased over these 17 years. Isoniazid resistance decreased from 12.4% in 1993–1996 to 10.2% in 2007–2009 (p<.001) and MDR TB decreased from 2.1% in 1993–1996 to 1.6% in 2007–2009 (p<.001).

When we limited NTSS data to sputum smear-positive cases for those who originated from countries in which case detection is based on microscopy, a total of 46,305 foreign-born TB cases were included in analysis. We included 14 individual countries as the unit of analysis, and grouped 208 countries/territories into one of nine WHO-defined TB epidemiologic regions. In recent cases (2007–2009) the highest rates of any isoniazid resistance were reported from Eastern Europe (18.0%) and South-East Asia (15.4%) regions. Of the 14 countries analyzed individually, the top 5 were Vietnam (19.2%), Peru (18.2%), the Philippines (15.6%), Dominican Republic (14.6%), and Republic of Korea (13.6%) (**Table 1**). During this same time period, the highest rates of MDR TB were reported from Eastern Europe (9.3%) and South-East Asia (4.7%) regions. Of the countries analyzed individually, the top 5 individual were Peru (5.8%), Dominican Republic (4.2%), Vietnam (2.3%), Ethiopia (2.0%), and Republic of Korea (1.9%) (**Table 1**).

The Global DRS data contained results from 207,987 new TB cases from 1994 to 2007. After excluding cases from the U.S. and the territories of the U.S., 172,015 cases remained. Of these, 14,784 (8.6%) had isoniazid resistance and 4,281 (2.5%) had MDR TB.

**Figure 1:**
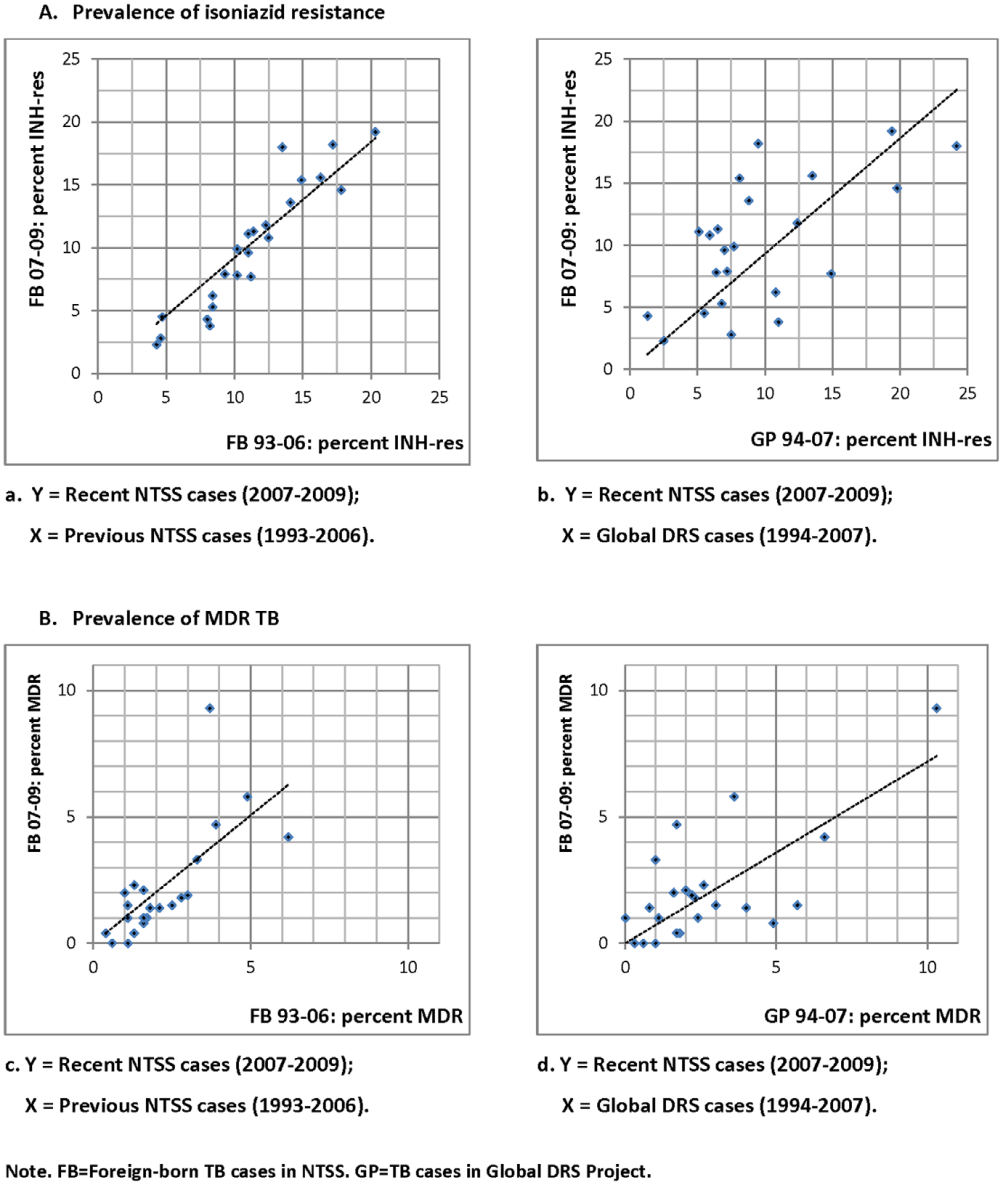
Scatterplots of (A) prevalence of isoniazid resistance among recent foreign-born NTSS cases (2007–2009) versus prevalence of isoniazid resistance among (a) previous foreign-born NTSS cases (1993–2006), and (b) Global DRS TB cases (1994–2007), and (B) prevalence of MDR TB among foreign-born NTSS cases (2007–2009) versus prevalence of MDR TB among (c) previous foreign-born NTSS cases (1993–2006), and (d) Global DRS TB cases (1994–2007). Each dot represents a country or a region as specified in Table 1.

Visual assessment of scatterplots showed that linear relationship for prevalence of both isoniazid resistance and MDR TB was much clearer for recent NTSS (2007–2009) versus previous NTSS (1993–2006) data than for recent NTSS (2007–2009) versus Global DRS (1994–2007) data (**Figure 1**). Furthermore, the recent NTSS data (2007–2009) on the prevalence of isoniazid resistant TB had stronger correlation, and better fit of linear regression model with previous years of NTSS data (1993–2006) (*r* = 0.95, *R* ^2^ = 0.87, *P*<.001) than with Global DRS data (1994–2007) from the corresponding countries or regions (*r* = 0.55, *R* ^2^ = 0.39, *P* = .001) (**Table 2**). NTSS data (2007–2009) on the prevalence of MDR TB had slightly stronger correlation, and slightly better linear regression model fit with previous years of NTSS data (1993–2006) (*r* = 0.75, *R* ^2^ = 0.55, *P*<.001) than with Global DRS data (1994–2007) from the corresponding countries or regions (*r* = 0.50, *R* ^2^ = 0.48, *P*<.001) (**Table 2**). Similar associations were found when making these comparisons in previous years **(** **Table 2** **)**.

**Table 2:** Correlation and Simple Linear Regression Analyses of Prevalence of Drug-resistant TB in Recent and Previous U.S. NTSS Data and Global DRS Data.

Outcome (Prevalence of Drug-resistant TB in MoreRecent NTSS Data)	Predictor (Prevalence of Drug-resistant TB in Previous NTSS orPrevious Global DRS Data)	Isoniazid Resistance				Multidrug Resistance			
		Correlation Coefficient (r)	Linear Regression Analysis			Correlation Coefficient (r)	Linear Regression Analysis		
			Slope (β_1_)	R^2^ Regression	P Value*		Slope (β_1_)	R^2^ Regression	P Value*
**NTSS (2007–2009)**	**NTSS (1993–2006)**	0.95	1.14	0.87	<.001	0.75	1.10	0.55	<.001
	**Global DRS (1994–2007)**	0.55	0.58	0.39	.001	0.50	0.64	0.48	<.001
**NTSS (2007–2009)**	**NTSS (2003–2006)**	0.89	1.12	0.81	<.001	0.79	1.39	0.80	<.001
	**Global DRS (2003–2006)**	0.65	0.60	0.45	.003	0.46	0.70	0.64	<.001
**NTSS (2003–2006)**	**NTSS (2000–2002)**	0.89	0.77	0.80	<.001	0.83	0.49	0.45	<.001
	**Global DRS (2000–2002)**	0.38	0.24	0.20	.09	0.40	0.38	0.45	.004
**NTSS (2000–2002)**	**NTSS (1997–1999)**	0.76	0.86	0.63	<.001	0.67	0.64	0.34	.004
	**Global DRS (1997–1999)**	0.75	0.50	0.49	.004	0.47	0.33	0.15	.15
**NTSS (1997–1999)**	**NTSS (1993–1996)**	0.68	0.62	0.49	<.001	0.62	0.55	0.38	.002
	**Global DRS (1994–1996)**	0.38	–	–	–	−0.05	–	–	–

Note. *P value is reported for F test of overall significance of simple linear regression model. Dashes were put for comparisons where no linear association was observed. NTSS = National Tuberculosis Surveillance System. Global DRS =  Global Project on Anti-Tuberculosis Drug Resistance Surveillance.

## Discussion

Treating TB infection or disease without DST results requires an educated guess as to the probability the patient is infected with a drug resistant strain of MTB. In the U.S., the recommended initial empiric treatment for active TB disease consists of four drugs (isoniazid, rifampin, ethambutol, and pyrazinamide), adequate to cover the possibility of isoniazid resistance, but inadequate for treatment of MDR TB. This regimen is recommended regardless of the patient’s country of birth. A substantial fraction of patients must be treated empirically for their entire course of therapy. For example, 20% of foreign-born TB cases in the U.S. were culture-negative and therefore could not have DST performed [1]. These considerations are especially important for LTBI because the number of LTBI cases is estimated to be 40-fold higher than the number of active cases (CDC unpublished data), LTBI is always treated empirically nearly always with a single drug, isoniazid. Isoniazid resistance is much more common than multidrug resistance [1].

Information on a country or region of origin is part of the epidemiological context of each case and may be used for clinical decision-making by U.S. healthcare providers along with the rest of the history and diagnostic evaluation. Further research should establishing numerical thresholds that would trigger specific management decisions. In the past, for example, the American Thoracic Society and the Centers for Disease Control and Prevention recommended adding fourth drug (ethambutol or, in young children, streptomycin) to the standard 3-drug regimen of isoniazid, rifampin, and pyrazinamide when the probability of isoniazid resistance exceeded 4% [9]. Given the wealth of information provided by the Global DRS, clinicians may turn to this resource for information about the prevalence of drug resistance in the country of birth of their particular patients [10]. While data on the prevalence of drug resistance may be useful for individualizing empiric treatment, they are likely to be most helpful in deciding whether to use rapid molecular tests for drug resistance [11]. For LTBI, the prevalence of isoniazid resistance could be considered among other factors when deciding between isoniazid or rifampin regimens [12].

Global DRS data, however, are based on representative sampling of TB cases in a particular country. U.S. foreign-born TB cases are not a representative sample of TB cases in their country of birth. Immigrants, business travelers, students, and tourists may be distributed across socioeconomic strata differently than the general population of their country of birth. The characteristics, including drug resistance, of TB in such foreign-born persons likely differs from that of TB cases occurring in their countries of birth. In our analysis we demonstrate that a better estimate of the prevalence of drug resistance among recent U.S. foreign-born TB cases can be obtained from NTSS data on country- or region-specific prevalence of drug resistance among previous foreign-born cases in the U.S. Conversely, despite suggestions that industrialized countries’ surveillance data on drug resistance prevalence in foreign-born TB cases could be used as an indicator of the prevalence of drug resistance in the patients’ countries or regions of birth, [13], [14] these data may not be appropriate because they are a biased sample.

Our analysis is subject to important limitations. We examined only prevalence of resistance to INH and RIF, and it is possible that our findings may not hold true for other drugs. Also, for those countries for which we had to aggregate the analysis by region, regional data may not be a good surrogate for individual countries within the region, given the large variation in drug resistance across countries within a region, restricting making clinical decisions for individuals from specific countries. This analysis examined correlations by aggregating data geographically as well as aggregating data over time. There may be specific subgroups of the foreign-born population, e.g., those who entered the country recently, as well as specific countries or regions for which Global DRS data reflect resistance patterns in the corresponding foreign-born persons in the US more accurately than US surveillance data. Similarly, it is possible that correlations with both Global DRS data and US surveillance data would improve, sample size permitting, if the databases were restricted to more recent years, e.g., after 2000, rather than including data from the way back to 1993 and 1994. We cannot exclude differences in the quality and accuracy of diagnostic methods used between USA and other countries. Finally, information about health, socio-economic and other relevant differences between persons who are US immigrants and visitors and the general populations of the country they emigrate from was not available in our data sources.

In summary, the prevalence of resistance to isoniazid and multidrug resistance among foreign-born TB cases in the U.S. is better predicted by the prevalence of drug resistance in previous foreign-born cases in the U.S. than by the prevalence of drug resistance among TB cases in their countries of origin. Thus, NTSS data are more likely to be useful than data from the Global DRS in guiding evaluation and management of TB in foreign-born patients in whom there is initial microbiologic evidence, epidemiological evidence, or a strong clinical suspicion of drug-resistant TB.
